# The use of Loop-mediated Isothermal Amplification (LAMP) to detect the re-emerging Human African Trypanosomiasis (HAT) in the Luangwa and Zambezi valleys

**DOI:** 10.1186/1756-3305-5-282

**Published:** 2012-12-04

**Authors:** Boniface Namangala, Lottie Hachaambwa, Kiichi Kajino, Aaron S Mweene, Kyouko Hayashida, Martin Simuunza, Humphrey Simukoko, Kennedy Choongo, Pamela Chansa, Shabir Lakhi, Ladslav Moonga, Amos Chota, Joseph Ndebe, Mutale Nsakashalo-Senkwe, Elizabeth Chizema, Lackson Kasonka, Chihiro Sugimoto

**Affiliations:** 1 Department of Paraclinical studies, School of Veterinary Medicine, University of Zambia, P.O. Box 32379, Lusaka, Zambia; 2 Department of Internal Medicine, University Teaching Hospital, PB RW 1X, Lusaka, Zambia; 3 Research Centre for Zoonosis Control, Hokkaido University, Kita-ku, Sapporo, 001-0020, Japan; 4 Department of Disease Control, School of Medicine, University of Zambia, P.O. Box 32379, Lusaka, Zambia; 5 Department of Biomedical Sciences, School of Medicine, University of Zambia, P.O. Box 32379, Lusaka, Zambia; 6 Ministry of Health, Ndeke House, P.O. Box 30205, Lusaka, Zambia

**Keywords:** HAT, RIME-LAMP, SRA-LAMP, *Trypanosoma brucei rhodesiense*, Zambia, Zimbabwe

## Abstract

**Background:**

Loop-mediated isothermal amplification (LAMP) is a novel strategy which amplifies DNA with high sensitivity and rapidity under isothermal conditions. In the present study, the performance of the repetitive insertion mobile element (RIME)-LAMP and human serum resistance-associated gene (SRA)-LAMP assays were evaluated using clinical specimens obtained from four male patients from Luangwa and Zambezi valleys in Zambia and Zimbabwe, respectively.

**Findings:**

The cases reported in this preliminary communication were all first diagnosed by microscopy, through passive surveillance, and confirmed by both RIME-LAMP and SRA-LAMP. A good correlation between microscopy and LAMP was observed and contributed to staging and successful treatment of patient. RIME-LAMP and SRA-LAMP complimented each other well in all the cases.

**Conclusions:**

Both RIME-LAMP and SRA-LAMP were able to detect *Trypanosoma brucei rhodesiense* DNA in patient blood and CSF and hence confirmed HAT in the parasitaemic patients. Our study indicates that the LAMP technique is a potential tool for HAT diagnosis, staging and may be useful for making therapeutic decisions. However, no statistically significant conclusion may be drawn due to the limited sample size used in the present study. It is thus imperative to conduct a detailed study to further evaluate the potential of LAMP as a bedside diagnostic test for HAT.

## Findings

Human African trypanosomiasis (HAT) or sleeping sickness is one of the neglected tropical diseases (NTDs) that mainly affect resource-poor countries in sub–Saharan Africa [1]. HAT is caused by *Trypanosoma brucei rhodesiense* (East and Southern Africa) and *Trypanosoma brucei gambiense* (West and Central Africa). It is transmitted through the bite of an infected tsetse fly (*Glossina* species) during a blood meal, with several domestic and wild animals acting as reservoirs of infection in *rhodesiense* HAT [1-3]. The disease comprises an early stage, characterized by the presence of parasites in blood and lymph and a late stage, characterized by parasite invasion of the central nervous system (CNS). In HAT endemic regions, routine disease diagnosis depends on clinical history of the patient, symptoms and mainly visualization of parasites in body fluids by microscopy [1,4]. However, HAT clinical symptoms are not pathognomonic and may be confused with those of other endemic febrile diseases. Although microscopy is associated with low sensitivity due to fluctuating parasitaemia in *gambiense* HAT patients, parasitological confirmation is relatively easier in *rhodesiense* HAT patients because bloodstream trypanosomes are numerous [4]. Molecular techniques such as PCR have higher sensitivity and specificity for trypanosome detection [5,6]. However, the cost implications and requirement for highly skilled manpower are obstacles to their wide application in clinical settings in sub-Saharan Africa. Consequently, diagnosis of HAT in endemic regions remains unsatisfactory.

Loop-mediated isothermal amplification (LAMP) is a novel strategy which amplifies DNA with high sensitivity and rapidity under isothermal conditions (60-65°C), producing large quantities of DNA within 30-60 minutes [7]. This allows visual detection of amplicons by naked eyes or through measurement of turbidity or fluorescence [8-10]. LAMP has been successfully developed and applied in the detection of various pathogens including protozoan parasites such as trypanosomes and *Babesia* species [11-13]. Advances in the development of trypanosome-specific LAMP have been made through the identification of conserved sequences (used to design highly specific LAMP tests) such as (i) the repetitive insertion mobile element (RIME) within the sub-genus *Trypanozoon*[6] and in particular (ii) the human serum resistance-associated (SRA) gene, which defines *T. b. rhodesiense* and therefore provides unequivocal identification of the parasite [5,14,15]. The RIME-LAMP and SRA-LAMP primers were previously designed by Njiru et al. [6,15].

In the present study, we evaluated the performance of RIME-LAMP and SRA-LAMP against routine microscopy, using clinical specimens obtained from four male HAT patients from the Luangwa and Zambezi river valleys in Zambia and Zimbabwe, respectively. The patient data is summarized in Table 1. Each of the HAT patients complained of a headache, dizziness, general malaise, intermittent fever, loss of appetite and fatigue. Clinical examination revealed pale mucous membranes, jaundice in some cases, enlarged lymph nodes, average body temperature of about 39.5°C. In addition, patients A - C exhibited impaired kidney function as evidenced by greatly increased creatinine levels (data not shown) while the HAT condition of patient D was less severe. All the patients sought medical attention at the University Teaching Hospital (UTH) in Lusaka, Zambia, where they were admitted for one to two months.

**Table 1:** Summary of patient data for some hospital reported HAT cases from the Luangwa and Zambezi valleys during the period 2010 – 2012

**Patient ID**	**Gender**	**Age**	**Occupation**	**Contracted HAT from**	**Month/Year Reported**
A	Male	Adult	Driver	Chama, near Mbambanda Sanctuary, Zambia	December 2010
B	Male	Adult	Telecommunication Company Employee	Chama, near Mbambanda Sanctuary, Zambia	January 2011
C	Male	Adult	Game Ranger	Mashonaland, Hurungwe Safari Area, Zimbabwe	January 2012
D	Male	Adult	Game Ranger	Mashonaland, Hurungwe Safari Area, Zimbabwe	May 2012

After clinical examination, patient blood and CSF samples were collected. The presence of trypanosomes was detected by microscopic examination of the buffy coat following centrifugation of blood and white blood cell (WBC) count in the case of CSF. The samples were then sent to the School of Veterinary Medicine, University of Zambia, for identification of trypanosome species by LAMP. About 200 μl of each sample (blood or CSF) was placed on a labeled FTA® Elute card (Whatman FTA® Elute Cards, Whatman, UK) for DNA extraction according to the manufacturer’s suggested protocol. The resultant DNA was stored at minus 30°C until use. A LAMP reaction of 25 μl was performed using a Loopamp DNA Amplification Kit (Eiken Chemical, Tochigi, Japan) and the extracted DNA as template, as described by Thekisoe et al. [11]. We used primers recently described by Njiru et al. [6,15] for the RIME-LAMP and SRA-LAMP, respectively. The reaction mixture was incubated at 64°C for 30 minutes in a heat block (Dry Thermounit DTU 1B, TAIEC Co., Saitama, Japan) and then at 95°C for 2 minutes to terminate the reaction. The LAMP products were visualized using a transilluminator (WD, H19, Good design award Co., Japan).

**Figure 1:**
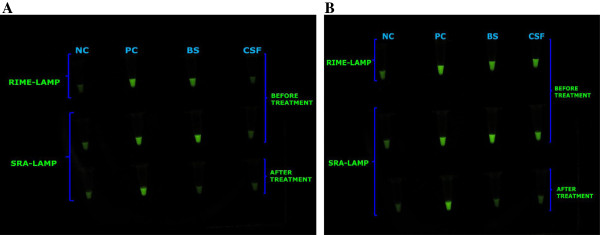
**Visual appearance of representative results for repetitive insertion mobile element (RIME)-LAMP and human serum resistance-associated gene (SRA)-LAMP before and after treatment of a patient with Early (A) and Late (B) Stage human African trypanosomiasis (HAT).** Loopamp Fluorescent detection reagent was added to the reaction mixture at the beginning of the assay. The reactions were incubated at 64°C for 30 minutes. In contrast to the light green background fluorescence in the negative samples, positive samples exhibit a bright fluorescent green colour when visualized under the transilluminator. NC: Negative control (distilled water); PC: Positive control (*Trypanosoma brucei rhodesiense*); BS: patient blood sample; CSF: patient cerebral spinal fluid sample.

The presence of trypanosomes in the HAT patients was confirmed by RIME-LAMP specific for the *Trypanozoon* subgenus group to which *T. brucei* complex belongs (Figure 1A-B). SRA-LAMP further identified the subspecies of the trypanosomes as the human-infective *T. b. rhodesiense* (Figure 1A-B). The presence of *T. b. rhodesiense* only in patient blood but not in CSF, coupled with ≤ 5 WBCs/mm^3^ in CSF, signified early stage HAT (Figure 1A, Table 2), which was treated with suramin [1] along with supportive therapy. On the other hand, the presence of the parasites in both blood and CSF, coupled with >5 WBCs/mm^3^ in CSF, indicated late stage HAT (Figure 1B, Table 2), which was treated with melarsoprol [1] along with supportive therapy. All the four treated HAT patients under study eventually recovered from the disease.

**Table 2:** Summary of clinical data for the HAT patients admitted to the University Teaching Hospital, Lusaka during the period 2010 – 2012

**Patient ID**	**Parasites in Blood**	**Parasites in CSF**	**HAT Stage**	**Treatment**	**Outcome**
A	Yes	No	Early	Suramin	Recovered
B	Yes	Yes	Late	Melasoprol	Recovered
C	Yes	No	Early	Suramin	Recovered
D	Yes	No	Early	Suramin	Recovered

Control of HAT heavily relies on accurate diagnosis and effective case management [1,16]. In the present study, we evaluated the performance of LAMP against microscopy, using clinical specimens obtained from four patients from Luangwa and Zambezi valleys. LAMP was not only sensitive, but also very specific [6,15] and confirmed the microscopic observation of the trypanosomes in patient blood and CSF. Four other HAT suspected cases from Chama that tested negative by microscopy also tested negative by LAMP (data not shown), demonstrating good concordance between the two methods. In agreement with Matovu et al. [17], RIME-LAMP and SRA-LAMP exhibited similar sensitivities to detect *T. b. rhodesiense* in the blood and CSF of HAT patients and complimented each other.

According to our preliminary results, LAMP confirmed the standard staging criteria (microscopy and WBC in CSF) [1,18,19], which led to successful treatment of all the patients. Accurate staging of HAT is critical for the therapeutic decisions as demonstrated in the present study. LAMP appears to be a potential tool for HAT staging and may thus prove to be a useful guide in making therapeutic decisions for HAT patients. However, in view of the fact that the presence of trypanosomes alone without any CSF alterations may be insufficient for second stage HAT diagnosis, HAT staging in this study also considered WBC count in the CSF. It is noteworthy, that detection of trypanosome DNA in CSF does not always signify active infection because of the potential for false positive cases resulting from dead parasites in the blood entering the CNS through the blood-brain-barrier or parasites that die in the CSF, in particular following drug therapy [18,19].

The four *rhodesiense* HAT cases reported in the present study were all detected through passive surveillance. It is possible that these cases could represent several others that may be unreported considering that HAT affects the poorest among the poor remote rural communities where social amenities are either weak or non-existent. According to Odiit et al. [20], about 39% of *rhodesiense* HAT cases and 92% of the deaths it causes are unreported. Indeed, there are several unpublished sporadic HAT cases reported at local health centers, mainly in the tsetse infested Luangwa and Zambezi valleys linked to National Parks [21]. Of note, in 2008 alone, between the months of March and July, about 12 cases were reported in Chama district along the borders of Zambia and Malawi. These cases were found amongst Zambia Wildlife Authority staff assigned to the then newly opened Mbambanda Zaro sanctuary [22]. Thus, in agreement with Mwanakasale and Songolo [23], there is need to increase both active and passive surveillance of HAT as well as community sensitization, particularly in HAT old foci where the disease appears to be re-emerging.

We envisage that this study will stimulate research to investigate the routine use of sensitive, specific and user-friendly techniques such as LAMP in the detection of emerging and re-emerging infectious diseases in endemic regions. Detailed studies using a larger sample size for further evaluation of the LAMP technique for diagnosis and staging of HAT patients, in particular in studies on HAT treatment responses, are justifiable.

### Ethical clearance

This study received ethical clearance for collection of human blood and cerebral spinal fluid from the Ministry of Health, Zambia, and from the Biomedical Research Ethics Committee, University of Zambia. Samples were collected after written informed consent was obtained from the participants in the presence of independent witnesses.

## Abbreviations

HAT: Humana African trypanosomiasis; NTDs: Neglected tropical diseases; T. b. rhodesiense: Trypanosoma brucei rhodesiense; PCR: Polymerase chain reaction; CNS: Central nervous system; LAMP: Loop-mediated isothermal amplification; RIME: Repetitive insertion mobile element; SRA: Human serum resistance-associated antigen; CSF: Cerebral spinal fluid; UTH: University teaching hospital.

## Competing interests

The authors declare that they have no competing interest.

## Authors’ contribution

NB helped to conceive the study, participated in its design, performed microscopy, purified the DNA from patient blood and CSF, performed the LAMP Assays, analyzed data and drafted the manuscript. HL, CP and LS were involved in the initial diagnosis of HAT and treatment of patients (HAT management), sample collection and helped in editing the manuscript. SM helped to conceive the study, participated in its design and helped in editing the manuscript. MAS, HK, SH, CK, NM, KL and CE helped to conceive the study and participated in its design. ML, CA and NJ performed microscopy, purified the DNA from patient blood and CSF and performed the LAMP Assays. SC and KK helped to conceive the study, participated in its design and assisted in obtaining funding. All the authors read and approved the final manuscript.
